# Sensory-Directed Identification of Creaminess-Enhancing Semi-Volatile Lactones in Crumb Chocolate

**DOI:** 10.3390/foods10071483

**Published:** 2021-06-25

**Authors:** Julia Samfaß, Timo D. Stark, Thomas F. Hofmann

**Affiliations:** Food Chemistry and Molecular Sensory Science, Technische Universität München, Lise-Meitner-Straße 34, 85354 Freising, Germany; lmc.julia@gmx.de (J.S.); thomas.hofmann@tum.de (T.F.H.)

**Keywords:** creaminess, milk chocolate, lactones, cocoa butter

## Abstract

In order to gain a more comprehensive knowledge of the chemical nature of creaminess-related flavor compounds in milk chocolates on a molecular level, crumb chocolate was analyzed by means of activity guided screening techniques. Sensory studies of a triglyceride-free lipid emulsion indicated that the n-pentane extract showed the highest impact regarding creaminess sensation. Enhancement of creaminess by adding anhydrous milk fat fractions to chocolate was demonstrated by fractionated high-vacuum distillation of different fats associated with the chocolate production combined with sensory experiments. Syntheses of various δ-lactones and the quantification of these sensory active semi-volatiles led to the conclusion that the anhydrous milk fat contains a series of γ- and δ-lactones. Cocoa butter revealed a high concentration of δ-hexadecalactone, too. Experiments suggested that lactones are generated from the potential precursors monohydroxyalkanoic acid(s) esterified (mono-tri)glyceride(s) during heating. Sensory studies exhibited recognition thresholds of 29–40 µmol/kg for the long-chain δ-lactones in crumb chocolate. Furthermore, significant enhancement of the retro-nasal creamy flavor was found for δ-tetradecalactone.

## 1. Introduction

The first chocolate bar was produced in 1874 by Fry & Sons. It was a combination of cocoa butter and non-pressed (full fat) cocoa and sugar. Adding dried milk to the chocolate, the Swiss innovator Henry Nestlé and Swiss chocolatier Daniel Peter became the inventors of the first milk chocolate. To improve the flavor and texture, Rudolph Lindt introduced a kneading process called conching [1,2].

Primarily, there are three chocolate categories: dark, milk, and white chocolate, differing in content of cocoa solid, milk fat, and cocoa butter. Different national consumer preferences and company practices resulted in variations in chocolate manufacturing processes [3].

Usually, milk chocolate is produced by two different manufacturing procedures. In the United Kingdom and other countries, creating a crumb is popular. One common method of making crumb is by combining the dry ingredients, primarily sugar, milk powder, and cocoa mass, with a small amount of water to form a paste, which is then dried in a high-vacuum oven at 75–105 °C to a moisture content of around 1% within 4–8 h. This crumb process was originally introduced to extend the storage life of milk powder, but is known to generate a typical unique flavor that consumers associate with this type of crumb chocolate. In the United Kingdom, crumb chocolate is known to be available in various flavors. Most chocolate bars, however, have some impression of cooked note with a high creaminess. Continental European milk chocolate is manufactured as a dry mix chocolate in which the crumb-making step is omitted and the dry ingredients are directly mixed with liquid fat [3,4].

The sensory impression “flavor” results from the simultaneous stimulation of the human chemical senses odor and taste, and is triggered by chemical compounds naturally present in food products. The consumers acceptance is strongly influenced by aroma-active volatiles as well as by non- or semi-volatiles, taste active compounds. “Creaminess” is a complex sensory attribute that is often considered as indicator of high quality and is a critical point with respect to consumers’ acceptance of many food products. As a sensory attribute, it has been used with several dimensions such as texture, flavor, and appearance [5]. Whenever consumers describe a product as creamy, certain sensory and hedonic characteristics are expected. Therefore, the term “creaminess” is a highly interesting and much debated topic [6]. Most published studies dealing with sensory evaluation define the term “creaminess” as a thick and smooth texture [7]. Tournier et al. conduct a sensory and a verbal approach to investigate the understanding of consumers’ creaminess concept. In this study, sensory properties of 12 dairy products were characterized by a trained panel. Then other consumers rated the creaminess and their liking for these products and expressed their own definition of the term “creaminess”. In summary, all consumers used words related to texture and pleasantness when defining creaminess. With respect to the attribute “creamy” the panelists created the following definition: “A product with a smooth texture, rather thick but which behaves like a fluid product when slightly pushing the tongue against the palate. Creaminess is not related to fatty dimension, a non-fat product can be creamy” [5,8]. As previously mentioned, the definitions of “creamy” mostly refer to texture requirements. However, “creaminess” has also been related to flavor perception, in particular to vanilla, sweetness, and fat-related flavors [5,6,9,10].

De Wijk et al. asked a trained panel to perform a sensory profile of model custard desserts varying in carrageenan, starch, and fat content. The results showed that the attribute “creamy/soft” was described as a combination with mouth-feel sensations (thickness and fattiness), after-feel sensations (fatty coating and absence of roughness), flavor/taste sensations (creamy and fatty flavors), and the absence of bitter/chemical and sickly flavors [9]. With regard to flavor, Kirkmeyer and Tepper conducted a free-choice profile of commercial dairy products (e.g., milk, cream, yogurt, ice cream). According to their results, the attributes “creamy texture” and “creamy flavor” were the opposites of the attributes “watery texture” and “watery”, “bland” flavor [11]. 

In the literature, the creaminess-inducing activity of δ-tetradecalactone is described to contribute to the retro-nasal aroma of whipped cream. Furthermore, γ- and δ-octadecalactone were found to evoke a significantly accelerated melting behavior in the oral cavity [12].

The objectives of this investigation included the identification of key components contributing to the creaminess of crumb chocolate besides the textural properties of the term creaminess. Furthermore, this study aimed to qualify which raw materials are responsible for enhancing the creaminess in chocolate and which technological processes are able to generate high concentrations of creaminess enhancers. The impact of key flavor components on the creaminess perception of crumb chocolate was evaluated by sensory experiments.

## 2. Materials and Methods

### 2.1. Fat and Chocolate Samples

Cocoa butter (CCB), anhydrous milk fat (AMF), and vegetable fat (VG) used for chocolate manufacturing were obtained from the chocolate industry. These batches of raw materials were used for producing the crumb and the crumb chocolate as well as for the dry mix chocolate in exactly the same proportions, and for the dairy-free chocolate. On the other hand, five milk chocolate samples and three milk-free chocolate samples were purchased at the German retail market.

### 2.2. Cocoa Liquor Samples

Cocoa liquor samples of different fermentation time, unroasted and low/high roasted, were obtained from the chocolate industry. The chocolate liquor was made of a low cocoa flavor bean, fermented via traditional box fermentation in Brazil.

### 2.3. Chemicals

The following compounds were obtained commercially: δ-tetradecalactone, calcium chloride (anhydrous, granular), potassium carbonate, chloroform (anhydrous), MCPBA (3-Chloroperoxybenzoic acid), ethyl-2-oxocyclopentanecarboxylate (Sigma-Aldrich, Steinheim, Germany); hydrochloric acid, acetic acid (glacial), formic acid, sodium hydrogen carbonate (Merck, Darmstadt, Germany); 1-bromoundecane, 1-bromotridecane, 2-bromododecane (Merck Suchardt OHG, Hohenbrunn, Germany); n-hexane, diethyl ether, n-pentane (Merck, Karlsruhe, Germany); potassium iodine (anhydrous), sodium chloride (Alfa Aesar, Karlsruhe, Germany); sodium sulfate (anhydrous), acetone (anhydrous) (VWR, Leuven, Belgium); ethyl acetate, dichloromethane (VWR International S.A.S, France); methanol, acetonitrile (gradient grade) (Baker J.T., Deventer, The Netherlands). γ-tetradecalactone, γ-hexadecalactone, and γ-octadecalactone were synthesized as reported in literature [12]. Sucrose, lactic acid, sodium chloride, tannic acid, oleic acid, stearic acid (Sigma-Aldrich), caffeine, and monosodium glutamate (Merck) were used for sensory panel training. Gum Arabic (Ph Eur, from acacia tree), highly refined mineral oil (embryo tested, suitable for mouse embryo cell culture, light oil (neat)), ethylenediaminetetraacetic acid (EDTA, bioUltra) (Sigma-Aldrich, Steinheim, Germany), whey protein (100% natural whey protein isolate) from Olimp, and Evian tap water (low mineralization 500 mg/L) were applied for sensory analysis. All solvents that were not of HPLC gradient were distilled prior to use. Deuterated solvents (CDCl_3_) were obtained from Euriso-Top (Gif-sur-Yvette Cedex, France). Water for High Performance Liquid Chromatography (HPLC) separation was purified by means of a Milli-Q water advantage A 10 water system (Millipore, Molsheim, France).

### 2.4. Syntheses of δ-Hexa- and δ-Octadecalactones 

The syntheses were conducted in three steps according to the procedures reported in literature with some modifications [13,14,15]. For the first step, ethyl-2-oxocyclopentanecarboxylate was alkylated (20 h) with bromoalkyl-derivatives in the presence of K_2_CO_3_ and KJ with refluxing in anhydrous acetone. Consequently, a two-necked round bottom flask was fitted with a condenser. Via the addition funnel, K_2_CO_3_ (29.0 mmol) and KJ (4.1 mmol) were added. Then, a solution of ethyl-2-oxocyclopentanecarboxylate (12.8 mmol) in anhydrous acetone (30 mL) was added via the addition funnel. After 10 min, a solution of 1-bromoundecane (12.9 mmol) (for the synthesis of δ-hexadecalactone) or 1-bromotridecane (for the synthesis of δ-octadecalactone) in anhydrous acetone (8 mL) was supplemented, and the mixture was rapidly brought to reflux by heating in a silicone oil bath (Carl Roth, Warm- und Kalthaltemittel, Rotitherm ^®^ M220, Karlsruhe, Germany). After refluxing (20 h), the mixture was cooled to room temperature, diluted with diethyl ether (50 mL), and filtered (Filter Papers, MN 615 ff ¼, Ø 185 mm, Macherey-Nagel, Germany). The filtrate was concentrated under reduced pressure and diluted with ether (50 mL). The organic phase was washed in a separating funnel with water and brine (2 × 100 mL), dried over Na_2_SO_4_, filtered, and concentrated under reduced pressure. The residue was chromatographed via medium pressure liquid chromatography coupled to an evaporative light scattering detector (MPLC–ELSD) (TelosTM flash columns, prepacked silica gel cartridges (80 g), n-hexane/ethyl acetate, gradient: 100–0 to 60–40) to give a pale yellow oil (product step 1, eluted at 14% ethyl acetate). Then, the same apparatus was used for the second step of the synthesis. To a mixture of the pale yellow oil ((6.44 mmol), product step 1) and HOAc (15 mL), concentrated HCl (25 mL) was added. The mixture was rapidly brought to reflux by heating in a silicone oil bath. After 48 h, the solvent was evaporated under reduced pressure by means of a rotary evaporator. The residue was diluted with saturated aqueous NaHCO_3_ solution (50 mL) and extracted with diethyl ether (3 × 50 mL). The combined organic layers were washed once in a separating funnel with saturated aqueous NaHCO_3_ solution and brine (50 mL) in excess, dried over Na_2_SO_4_, filtered, and concentrated at reduced pressure. The target compound was purified via HPLC–ELSD and confirmed as 2-un-(or tri-)decylcyclopentane-1-one (product step 2) via ^1^H NMR spectroscopy. The third step was a Baeyer–Villiger reaction with MCPBA, therefore, a solution of the intermediate product ((0.23 mmol), product step 2) was re-suspended in anhydrous chloroform (3 mL) and was treated with NaHCO_3_ (0.34 mmol) and MCPBA (0.35 mmol). The mixture was stirred at room temperature for 8 h, diluted with saturated aqueous NaHCO_3_ solution (50 mL), vigorously stirred for 15 min, and extracted with dichloromethane (50 mL) in a separating funnel. The combined organic layers were washed with water and brine (50 mL) in excess, dried over Na_2_SO_4_, filtered, and concentrated at reduced pressure. The residue was chromatographed via MPLC–ELSD (TelosTM Flash columns, pre-packed silica gel cartridges (80 g), n-hexane/ethyl acetate, gradient: 100–0 to 60–40) to afford the corresponding title compounds as colorless oils at 30% ethyl acetate: δ-hexadecalactone, δ-octadecalactone.

#### 2.4.1. δ-(7-Methyl)-Hexadecalactone

As this δ-lactone does not naturally occur in chocolate, it was utilized as internal standard and synthesized according to literature with some modifications [13,14,15]. As reactant, 2-bromododecane was applied. The structure elucidation of all synthesized lactones was performed by means of ultra performance liquid chromatography coupled to time-of-flight mass spectrometry (UPLC/TOF-MS) in ESI+ and ESI− mode as well as 1D- and 2D-NMR experiments. The purity was determined by q-H NMR spectroscopy [16].

#### 2.4.2. δ-Hexadecalactone

UPLC/TOF-MS (ESI−): *m*/*z* 271.2274 (M+H_2_O-H)^−^, 271.2273 calculated for C_16_H_31_O_3_. (ESI+): *m*/*z* 277.2143 (M+Na)^+^, 277.2143 calculated for C_16_H_31_O_2_Na; *m*/*z* 237.2221 (MH_2_O)^+^, 237.2218 calculated for C_16_H_29_O; *m*/*z* 219.2213 (M 2xH_2_O)^+^, 219.2218 calculated for C_16_H_27_. Yield 5% (0.66 mmol); purity 98% via q-H NMR; ^1^H NMR (400 MHz, CDCl_3_, COSY): δ 0.88 (t, 3H, J = 6.86 Hz, H-C(16)), 1.20–1.35 (m, 8 × 2H, H-C(15)-H-C(8)), 1.35–1.50 (m, 2H, H C(7)), 1.50–1.62 (m, 2H, H-C(6b), H-C(4b)), 1.65–1.76 (m, 1H, H-C(6a)), 1.77–1.96 (m, 3H, H C(3), H-C(4a)), 2.38–2.63 (m, 2H, H-C(2)), 4.23–4.33 (m, 1H, H-C(5)). ^13^C NMR (100 MHz, CDCl_3_, HSQC, HMBC): δ 14.08 (C(16)), 18.54 (C(3)), 22.71 (C(15)), 24.92 (C(7)), 27.80 (C(4)), 29.34–29.64 (7xC, C(8)-C(13),C(2)), 31.94 (C(14)), 35.85 (C(6)), 80.64 (C(5)), 171.95 (C(1)).

#### 2.4.3. δ-Octadecalactone

UPLC/TOF-MS (ESI−): m/z 299.2584 (M+H_2_O-H)^−^, 299.2584 calculated for C_18_H_35_O_3_. (ESI+): m/z 305.2460 (M+Na)^+^, 305.2456 calculated for C_18_H_34_O_2_Na; m/z 265.25.30 (M-H_2_O)^+^, 265.2531 calculated for C_18_H_33_O; m/z 247.2424 (M 2xH_2_O)^+^, 247.2426 calculated for C_18_H_31_. Yield 5% (0.66 mmol); purity 98% via q-H NMR; ^1^H NMR (400 MHz, CDCl_3_, COSY): δ 0.88 (t, 3H, J = 6.65 Hz, H-C(18)), 1.16–1.35 (m, 10 × 2H, H-C(17)-H-C(8)), 1.35–1.51 (m, 2H, H C(7)), 1.51–1.62 (m, 2H, H-C(6b), H-C(4b)), 1.62–1.76 (m, 1H, H-C(6a)), 1.76–1.96 (m, 3H, H C(3), H-C(4a)), 2.38–2.63 (m, 2H, H-C(2)), 4.23–4.31 (m, 1H, H-C(5)). ^13^C NMR (100 MHz, CDCl_3_, HSQC, HMBC): δ 14.16 (C(18)), 18.52 (C(3)), 22.68 (C(17)), 24.90 (C(7)), 27.79 (C(4)), 29.36–29.69 (9xC, C(8)-C(15),C(2)), 31.95 (C(16)), 35.86 (C(6)), 80.66 (C(5)), 171.83 (C(1)).

#### 2.4.4. δ-(7-Methyl)-Hexadecalactone

UPLC/TOF-MS (ESI−): m/z 285.2428 (M+H_2_O H)^−^, 285.2428 calculated for C_17_H_33_O_3_. (ESI+): m/z 291.2301 (M+Na)^+^, 291.2301 calculated for C_17_H_32_O_2_Na; m/z 251.2372 (M-H_2_O)^+^, 251.2372 calculated for C_17_H_30_O; m/z 233.2268 (M 2xH_2_O)^+^, 233.2268 calculated for C_17_H_28_. Yield 0.2% (0.05 mmol); purity 95% via q-H NMR; ^1^H NMR (400 MHz, CDCl_3_, COSY): δ 0.88 (t, 3H, J = 6.86 Hz, H-C(16)), 1.20–1.35 (m, 8 × 2H, H-C(15)-H-C(8)), 1.35–1.50 (m, 2H, H C(7)), 1.50–1.62 (m, 2H, H-C(6b), H-C(4b)), 1.65–1.76 (m, 1H, H-C(6a)), 1.77–1.96 (m, 3H, H C(3), H-C(4a)), 2.38–2.63 (m, 2H, H-C(2)), 4.23–4.33 (m, 1H, H-C(5)). ^13^C NMR (100 MHz, CDCl_3_, HSQC, HMBC): δ 14.08 (C(16)), 18.54 (C(3)), 22.71 (C(15)), 24.92 (C(7)), 27.80 (C(4)), 29.34–29.64 (7xC, C(8)-C(13),C(2)), 31.94 (C(14)), 35.85 (C(6)), 80.64 (C(5)), 171.95 (C(1)).

### 2.5. Solvent Extraction of Chocolate and Cocoa Liquor

An aliquot of each sample (chocolate: 50 g; cocoa liquor: 5 g) was frozen in liquid nitrogen and ground in a laboratory blender (Messermühle, GRINDOMIX 300, Retsch Laborgeräte, Germany). The extraction was conducted for 30 min with n-pentane (fraction I, 3 × 100 mL), ethyl acetate (fraction II, 3 × 100 mL), and methanol/water (70/30 vol%, fraction III, 3 × 100 mL) at room temperature on a magnetic stirrer. After centrifugation (Zentrifuge, Beckman Coulter Avanti J-E, Germany) and filtration (Filter Papers, MN 615 ff ¼, Ø 185 mm, Macherey-Nagel), the procedure was repeated in triplicate. The solvents of the combined organic layers were removed in a vacuum, freeze-dried (48 h), and then stored at 20 °C until use. 

### 2.6. Isolation of Semi-Volatiles from Fat

An aliquot (32 g cocoa butter; yield of 100 g chocolate) of CCB, AMF, and VG, and the n-pentane extractables (fraction I) from the crumb chocolate, were vigorously stirred in a round bottom flask (250 mL) connected to a high-vacuum sublimation device (10^−3^ mbar) for 30 min at 50 °C to remove the volatiles without cold trapping. After that, the temperature was increased stepwise to 80, 120, 150, and 180 °C, and the fat was thermally treated in a silicone oil bath for one hour. The semi-volatile compounds released were cryofocused by means of a trap cooled by a cryostat to 0 °C. After one hour, the trapped compounds were rinsed off with n-pentane, and the temperature was increased to the next step. The solvent was evaporated and each generated fraction was freeze-dried for sensory experiments.

### 2.7. Sub-Fractionation of Fraction I

The n-pentane extractables from the chocolate/cocoa liquor (fraction I) were homogenously mixed with acetonitrile and stored at 20 °C for 24 h to freeze out the triglycerides (fraction Ib). After filtration (Filter Papers, MN 615 ff ¼, Ø 185 mm, Macherey-Nagel), the acetonitrile extractables without the triglycerides (fraction Ia) were evaporated from the solvent. Both sub-fractions were freeze-dried and stored at −20 °C until use for sensory experiments. 

### 2.8. Sensory Analyses

Panel training. Twelve assessors were recruited from the Chair of Food Chemistry and Molecular Sensory Science who gave informed consent to participate in the sensory tests, had no history of known taste disorders, and were familiar with the applied techniques. The panelists were trained to recognize and quantify the taste of aqueous solutions (3 mL each) of the following standard compounds dissolved in bottled water (Evian; low mineralization, 500 mg/L) by use of a triangle test: sucrose (50 mmol/L) for sweet taste, lactic acid (20 mmol/L) for sour taste, NaCl (15 mmol/L) for salty taste, caffeine (1 mmol/L) for bitter taste, monosodium glutamate (3 mmol/L) for umami taste, and gallustannic acid (0.05%) for astringency. Besides these, aqueous emulsions of a triglyceride-free lipid emulsion (TFLE) were used containing oleic acid (1 mmol/L) for fatty mouth feeling, stearic acid (1 mmol/L) for grainy, powdery mouth feeling, and δ-tetradecalactone (40 µmol/L) for creamy flavor impression. The TFLE contained gum Arabic (3%, Ph Eur, from acacia tree), ethylenediaminetetraacetic acid (0.01%, EDTA), highly refined mineral oil (1%, white oil), and whey protein (0.3%, 100% natural whey protein isolate) in table water (Evian) and was ultrasonificated at 40 °C for profile analysis [17]. Sensory analyses were performed in a sensory panel room at 22–25 °C in three different sessions.

#### 2.8.1. Precaution Taken for Sensory Analysis of Food Fractions and Synthesized δ-Lactones

To remove solvent traces from all fractions and synthesized δ-lactones, the individual fractions/syntheses treated with high vacuum by rotary evaporator (Rotavapor, BÜCHI, Germany). Afterwards, they were suspended in water and freeze-dried twice. ^1^H NMR spectroscopy and LC–MS/MS analysis of an aliquot revealed that chocolate/cocoa liquor fractions and syntheses treated with this procedure were essentially free of the solvents and other chemicals used for syntheses.

#### 2.8.2. Sensory Profiling of Chocolate and Isolated Chocolate Fractions in TFLE

The crumb chocolate and the isolated fractions (fraction I, II, III) were suspended in TFLE to get rid of the textural impressions and differences in the melting behavior. The chocolate itself (20 g/100 mL TFLE) and the three isolated fractions (fraction I, II, III) were suspended in their “natural” concentration ratios according to 20 g of chocolate in TLFE and presented to the sensory trained panel. The assessors evaluated the samples by rating the intensity of the sensory descriptors sweet, bitter, astringent, sour, fatty, creamy, and caramel on a scale from 0 (none at all) to 5 (extremely strong) without wearing a nose clip, to include the retro-nasal flavor impression after swallowing. The samples (2 mL) were briefly swirled in mouth before swallowing. After each tested fraction, the panelists had to rinse their mouths with Evian containing 2% ethanol. 

#### 2.8.3. Two-Alternative Forced-Choice Test (2-AFC Test)

Tests were carried out according to § 64 LFGB, method 00.90–19 with a constant reference. This method was used to study the difference in flavor of two samples. To evaluate the creamy intensity of the fat-distillation fractions, they were suspended in their “natural” concentration ratios according to 20 g of chocolate in TFLE and presented to the sensory trained panel in brown glasses. One glass was designated as “reference” and contained only the TFLE in pure form. The other two glasses were labeled with three-digit codes. One of the two samples contained only TFLE, the other one TFLE and the corresponding distillate.

To determine the recognition thresholds of δ-lactones in crumb chocolate three small plates (1 ± 0.1 g) were presented to the panelists. One small plate, containing the reference sample, was designated as “reference”, while the other two small plates were labeled with three-digit codes: one of the two samples contained the reference chocolate, and the other, the chocolate including a δ-lactone. Using a forced-choice methodology, the assessors had to identify the differing sample. Each sensory experiment was repeated three times, and the data is given as means of replicates.

#### 2.8.4. Recognition Thresholds of δ-Lactones in Crumb Chocolate

Ethanoic solutions of each purified δ-lactone were prepared (concentration 100 µmol/mL). The crumb chocolate was melted in a water bath at 40 °C and to the reference chocolate (chocolate without lactone added) as well as to the spiked chocolate (spiked with the ethanoic solution of lactone) the same aliquot of ethanol was added. Concentrations of δ-lactones varied from 20–100 µmol/kg chocolate, and for each concentration of lactone, an individual reference chocolate was prepared. Small plates of chocolate were put into plastic lids and cooled in the freezer to 5 °C before they were presented to the panelists. The panel was instructed to put the plate onto the tongue, press the tongue on the palate, and let it slowly melt in the mouth. After swallowing, each panelist was asked to determine the threshold concentrations for a change in mouth-feeling and retro-nasal perception, respectively. Following this two-alternative forced-choice protocol, two samples were compared to the reference chocolate. Between two tastings, the panelists had to rinse their mouth with an ethanolic solution (2%) in Evian. 

### 2.9. Identification and Quantification of δ-Lactones

#### 2.9.1. Identification of δ-Lactones in Fraction Ia

Schlutt et al. demonstrated the occurrence of long-chain lactones in whipped cream; therefore, the “fat fraction” (n-pentane extractables, fraction I) of the chocolate was further investigated [12]. Fraction Ia, obtained from 5 g chocolate/cocoa liquor after freezing out the triglycerides from the n-pentane extractables, was cleaned up for LC–MS/MS screening by solid phase extraction (SPE). Therefore, the SPE column (RP 18, Strata^®^ C18 E, octadecyl-modified silica, endcapped, 1 g/6 mL, Phenomenex, Aschaffenburg, Germany) was activated with acetonitrile (2 × 6 mL) and conditioned with 10% acetonitrile in water (2 × 6 mL). Then, fraction Ia (1 mL) was placed onto the top of the column, and a washing step with 10% acetonitrile in water (6 mL) was applied. Elution was performed with acetonitrile (6 mL). Following a software-assisted tuning of the reference compounds, fraction Ib was freeze-dried and re-suspended in acetonitrile for HPLC–MS/MS analyses (dilution 1:2 for chocolate, 1:1 for cocoa liquor) and screened for the occurrence of δ-lactones. 

#### 2.9.2. Quantification of δ-Lactones in Fraction Ia

Before extracting the chocolate or the cocoa liquor with n-pentane, the molten (40 °C, water bath) samples were spiked with a solution of δ-(7-methyl)-hexadecalactone as internal standard (4 µg for 5 g chocolate, cocoa liquor) in acetonitrile (0.1 mg/mL), homogenized, frozen with liquid nitrogen, and ground to a fine powder. After freezing out the triglycerides of fraction I (n-pentane extractables), SPE purification of fraction Ia (n-pentane extractables without triglycerides) and membrane filtration of the re-suspended fraction Ia (100% acetonitrile) were performed. Aliquots (4 µL) were analyzed by means of HPLC–MS/MS, which was equipped with a 250 × 4.6 mm i.d., 5 µm, RP Hyperclone column (Phenomenex) operated with a flow rate of 0.4 mL/min. Separation was performed, starting with 50% aqueous formic acid (0.1%) and 50% acetonitrile with formic acid (0.1%) for 1.5 min. The acetonitrile content was successively increased to 70% in 3.5 min, to 83% in 13 min, to 100% in 15 min, and finally held at 100% for 9 min. By means of the multiple reaction monitoring (MRM+) mode, δ-tetradecalactone (*m*/*z*: 227.2 → 191.1), δ-hexadecalactone (m/z: 255.3 → 237.3), and δ-octadecalactone (*m*/*z*: 283.3 → 265.3) were analyzed using the mass transitions given in the brackets and were monitored for a duration of 30 ms. The synthesized δ-(7-methyl)-hexadecalactone (*m*/*z*: 269.3 → 251.2) was used as internal standard for the quantitative analyses of all three lactones. Five mg of each δ-lactone were combined to give a stock solution. For calibration, the stock solution was diluted to concentrations ranging from 7.8 ng/mL to 100 µg/mL and, respectively 4 µg internal standard was added. After linear regression analysis of peak area versus concentration, the calibration curve showed a linear response (δ tetradecalactone: y = 0.1258x + 0.0601; δ-hexadecalactone: y = 0.4179x + 0.274; δ octadecalactone: y = 0.9713x + 0.0885) with a correlation coefficient of R^2^ > 0.95 for each δ-lactone. Samples were analyzed in biological as well as technical duplicates; standard deviation (SD) of the method was less than 10%.

### 2.10. Liquid Chromatography–Mass Spectrometry (LC–MS/MS)

LC–MS/MS analysis was performed using a Dionex UHPLC UltiMate^®^ 3000 HPLC system (Dionex, Idstein, Germany), which was connected to the API 4000 QTRAP linear ion trap quadrupole mass spectrometer (Applied Biosystems, Darmstadt, Germany) running in positive ionization mode (+5500 V). Both quadrupoles operated at unit mass resolution, and nitrogen served as nebulizer gas (nitrogen, 55 psi), turbo gas (nitrogen, 65 psi), and curtain gas (nitrogen, 35 psi). Data acquisition and instrumental control was performed with Analyst 1.6.2 software (Applied Biosystems, Darmstadt, Germany). 

### 2.11. UPLC/Time-of-Flight Mass Spectrometry (UPLC/TOF-MS)

High-resolution mass spectra of the synthesized δ-lactones were measured on a SYNAPT G2-S HDMS (Waters UK Ltd., Manchester, UK) in negative and positive ESI and high-resolution mode using the following parameters: capillary voltage +2.0 kV, sampling cone 30, source temperature 120 °C, desolvation temperature 450 °C, cone gas 30 L/h, and desolvation gas 850 L/h. All samples were dissolved in 1 mL acetonitrile, and aliquots (1–5 µL) were injected into the UPLC/UV/TOF-MS system. The samples were introduced into the instrument via an Acquity UPLC core system (Waters, Milford, MA, USA) consisting of a binary solvent manager, a sample manager, and a column oven. For chromatography, a 2 × 150 mm i.d., 1.7 µm, BEH C18 column (Waters) was operated with a flow rate of 0.4 mL/min at a temperature of 50 °C. The solvent system consisted of acetonitrile with formic acid (0.1% in acetonitrile, A) and aqueous formic acid (0.1% in water, B). The following gradient was used: 0 min/20% B, 4 min/100% B, 4.5 min/100% B, 4.6 min/20% B. The instrument was calibrated over m/z range of 50 to 1200 using a solution of sodium formate (0.5 mM). Data acquisition and interpretation were performed using MassLynxTM software Version 4.1 (Waters) and the tool “elemental composition”.

### 2.12. Nuclear Magnetic Resonance Spectroscopy (NMR)

One- and two-dimensional ^1^H and ^13^C NMR spectra were acquired on a 400 MHz DRX (equipped with a Z-gradient 5 mm multinuclear observe probe (BBFOplus)) and a 500 MHz Avance III spectrometer (equipped with a cryo-TCI probe) (Bruker, Rheinstetten, Germany), respectively. Deuterated chloroform (CDCl_3_, Sigma) was used as solvent, and chemical shifts are reported in parts per million relative to the solvent signal. For structural elucidation and NMR signal assignment, 2D NMR experiments such as COSY-, HSQC-, and HMBC-spectroscopy were conducted using the pulse sequences from the Bruker software library. Data processing was performed with TopSpin Version 3.0/3.2 (Bruker). Data analysis was done by NMR Software MestReNova-10.0.2-15465©2015 Mestrelab Research S.L. (Santiago de Compostela University, Spain). Q NMRs were measured by means of quantitative ^1^H NMR spectroscopy using L-tyrosine (3.03 mmol/L, D2O/DCl) and caffeine (3.12 mmol/L, D2O) as external standards [16].

## 3. Results and Discussion

**Figure 1:**
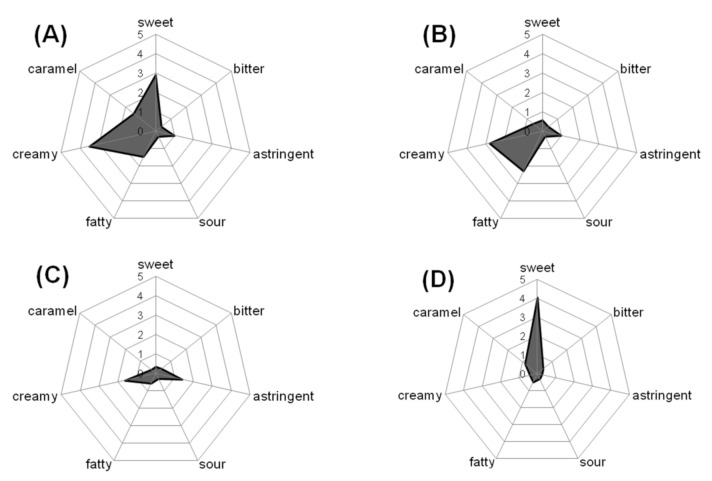
Sensory profiles of (**A**) crumb chocolate, (**B**) fraction I (n-pentane extract) of crumb chocolate, (**C**) fraction II (ethyl acetate extract) of crumb chocolate, and (**D**) fraction III (methanol/water 70/30 *v*/*v* extract), all suspended in triglyceride-free lipid emulsion (TFLE).

Crumb chocolate is known to be a type of chocolate with a very creamy flavor. An activity-guided solvent fractionation with solvents of increasing polarities was performed to localize the creamy flavor in crumb chocolate. A sensory profiling of chocolate and the obtained fractions was carried out by a sensory trained panel. To exclude textural differences, TFLE was used. The suspended crumb chocolate exhibited a strong creamy flavor. Additionally, a very sweet and caramel-like taste and a light fatty impression was recognized, whereas a bitter, sour taste and astringency was only perceptible in low intensities (Figure 1A). Comparing the three obtained extracts, the strongest intensity for the attribute creamy was observed in fraction I (Figure 1B), with minor intensity remaining in fraction II (Figure 1C). Fraction III induced only a very strong sweet and caramel taste (Figure 1D). Consequently, the creamy flavor was localized in fraction I and fraction II. Therefore, these fractions were further investigated. To exclude the influence of triglycerides in creaminess perception, fraction I was sub-fractionated by freezing out the triglycerides. The n-pentane extractables without triglycerides were obtained as fraction Ia. Approximately the same sensory profile was obtained by sensory evaluation of fraction Ia (data not shown) as mentioned above for fraction I. This led to the conclusion that triglycerides do not have an effect on creaminess perception; therefore, other components extractable with n-pentane probably evoke the creaminess perception. With regard to literature, long-chain fatty acid lactones found in non-heated cream and high-treated cream also might be detectable in chocolate, because AMF is one characteristic ingredient of crumb chocolate. Furthermore, it has been described that thermally treated cream, in comparison to non-heated cream, induced an increase of the creamy note in sensory profile analysis [12].

### 3.1. Identification of Sensory Active Semi-Volatiles

**Figure 2:**
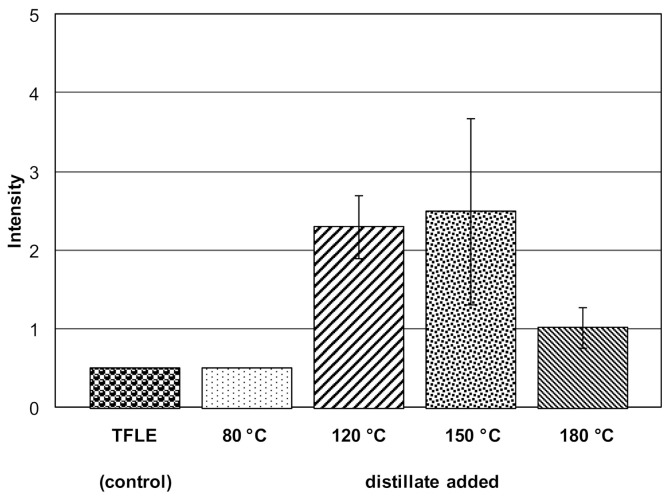
Enhancement of creamy flavor by the semi-volatile isolate obtained by *n*-pentane extraction at 80/120/150/180 °C. The isolates (natural concentration rations according to 20 g of chocolate per 100 mL TFLE) were suspended in TFLE for sensory evaluation.

In order to both identify the compounds responsible for the creamy flavor and to gain insights into the formation pathways of creaminess-enhancing molecules, a high-vacuum distillation with different fats used in chocolate manufacturing as well as with the n-pentane extract (fraction I) under heat treatment was performed. First, the volatiles were removed from the corresponding fat or fraction I by high-vacuum distillation. Then, the temperature was increased stepwise, and after 1 h, the semi-volatiles were trapped by means of a cryofocusing device. In the case of 120, 150, and 180 °C, the assessors were able to distinguish between the control (TFLE) and the spiked TFLE with the distillates of fraction I. In contrast, the 80 °C distillate added to TFLE could not be distinguished from the control. By far the highest creamy flavor could be observed at 120 and 150 °C. At 180 °C, a decrease of creaminess was detected by the panelists without nose clips (Figure 2). These data clearly demonstrate that semi-volatiles isolated from chocolate fat increase creaminess perception. The same experiment was performed with the high-vacuum distillates of the AMF, CCB, and VF (fats used for chocolate manufacturing) (Table 1). By adding the semi-volatile fractions of the CCB to TFLE, only a rancid flavor at 120 °C and a nutty flavor at 150 °C were detectable. With the fractions of AMF suspended in TFLE, the panelists perceived a strong creamy flavor for the distillates at 120, 150, and 180 °C, and a slight creamy flavor at 80 °C. In contrast, the distillates of VF did not show a creaminess-enhancing effect.

**Figure 3:**
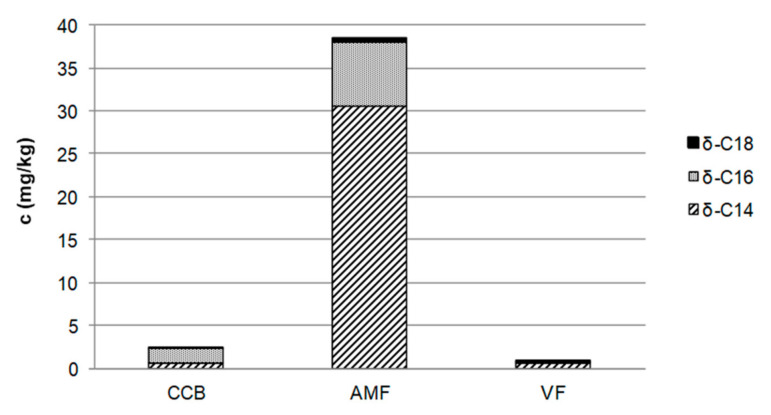
Concentrations of *δ*-tetra- (*δ*-C14), *δ*-hexa- (*δ*-C16), and *δ*-octadecalactone (*δ*-C18) in cocoa butter (CCB), anhydrous milk fat (AMF), and vegetable fat (VF) after removal of triglycerides determined by means of LC–MS/MS. (SD < 10%).

**Figure 4:**
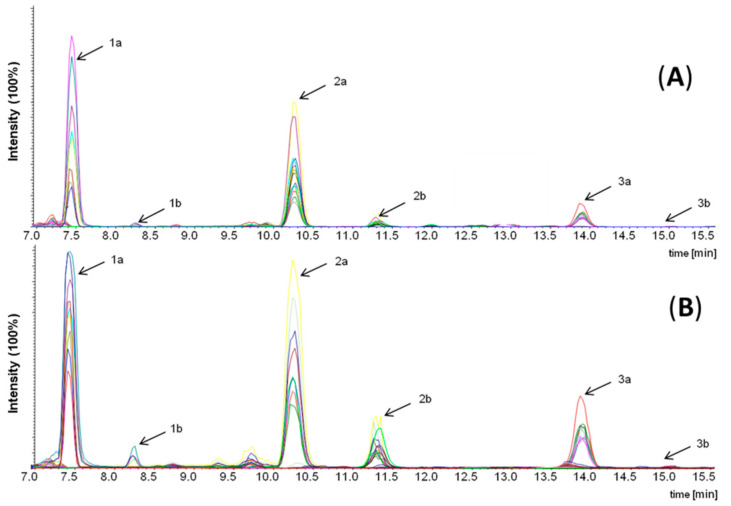
Reversed phase HPLC–MS/MS chromatograms of (**A**) anhydrous milk fat before high-vacuum distillation (acetonitrile solubles) and (**B**) semi-volatile isolates obtained by high-vacuum distillation of the anhydrous milk fat at 150 °C (acetonitrile solubles): 1a, *δ*-tetra-(*δ*-C14); 1b, *γ*-tetradecalactone (*γ*-C14); 2a, *δ*-hexa-(*δ*-C16); 2b, *γ*-hexadecalactone (*γ*-C16); 3a, *δ*-octa- (*δ*-C18); 3b, *γ*-octadecalactone (*γ*-C18).

Long-chain lactones are known to be sensory active creaminess enhancers in (whipped) cream [12]. Thus, it can be assumed that lactones might occur in high concentrations in the semi-volatile isolates of fraction I and the different fats. To prove this assumption, δ-hexadeca- and δ-octadecalactone were synthesized and their structures verified via MS as well as 1/2D NMR spectroscopy together with the commercially available δ-tetradecalactone used to tune the mass spectrometer. To speed up the identification of the active principle, the isolate with the highest sensory impact regarding creaminess was analyzed via LC–MS/MS operating in the ESI mode. The presence of these lactones was confirmed in fraction Ia in lower concentrations than in the distillates obtained by high-vacuum distillation of fraction I (data not shown). The fat extracted from the crumb chocolate consisted of the three fats used for chocolate manufacturing, i.e., CCB, AMF, and VF. Therefore, in order to clarify the contribution of each fat to the lactone content of the crumb chocolate, the concentration of lactones was determined in the individual fats (Figure 3). The highest total concentration of 38.5 mg/kg of the quantified δ-lactones was found in AMF, of which 79% originated from δ-tetradecalactone alone. In VF, only a very low concentration of lactones was determined, and CCB contained a detectable concentration of only δ-hexadecalactone (Figure 3). On the basis of these data, it can be concluded that AMF showed the highest contribution to the content of δ-lactones in chocolate. Earlier reports confirmed the presence of these lactones in heated butter fat, milk fat, and cheese, whereas no data of δ-lactones in CCB is available [18,19,20]. Furthermore, the potential of the different fats to liberate lactones was investigated in order to verify the kitchen-like formation pathways reported in literature and in order to identify possible food processing methods for enhancing the lactone concentration [18,20,21]. Therefore, all three fats used for chocolate manufacturing (AMF, CCB, VF) were distillated by means of a high-vacuum sublimation device at various temperatures (80, 120, 150, 180 °C). LC–MS/MS analyses obtained for AMF (acetonitrile solubles) before (Figure 4A) and after high-vacuum distillation at 150 °C (Figure 4B) showed a significant increase in the concentrations of all three δ-lactones 1a-3a. The corresponding γ-lactones 1b-3b could be separated from the δ-lactones by chromatography and were only detectable in traces in the high-vacuum distillation fraction at 150 °C of AMF. Thus, the γ-lactones were not quantified with the method described above.

**Figure 5:**
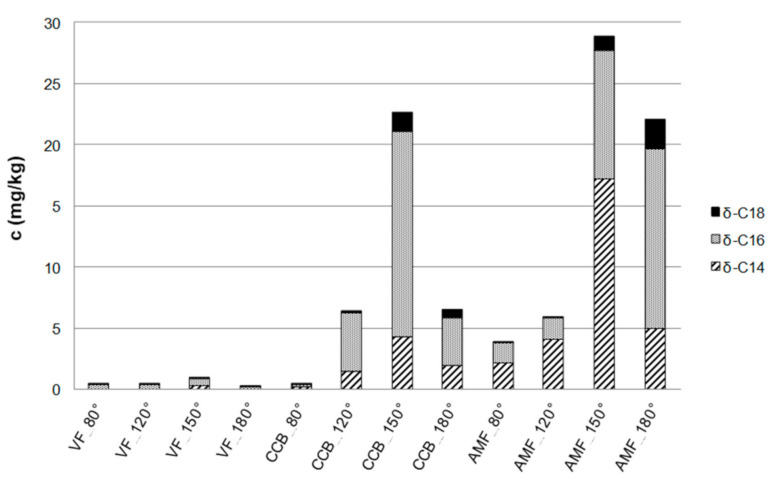
Concentrations of *δ*-tetra- (*δ*-C14), *δ*-hexa- (*δ*-C16), and *δ*-octadecalactone (*δ*-C18) in the semi-volatile isolate obtained by high-vacuum distillation at 80/120/150/180 °C of cocoa butter (CCB), anhydrous milk fat (AMF), and vegetable fat (VF) after removal of triglycerides determined by means of LC–MS/MS. (SD < 10%).

Subsequently, the semi-volatile isolates of the different fats were collected and the three δ-lactones were quantified (Figure 5). By comparing the total contents of δ-lactones in every semi-volatile fraction, it was ascertained that by high-vacuum distillation of VF in all four temperature steps, only traces of δ-lactones were released. In the cases of CCB and AMF, high concentrations of δ-lactones were especially obtained at 150 °C. δ-hexadecalactone showed the highest contribution for the semi-volatile fraction of CCB and δ- tetradecalactone for the AMF (Figure 5). In earlier reports, the lactone concentration in AMF was described as off-flavor of stored forms of milk that, therefore, could be distinguished from fresh milk [22,23,24]. In order to avoid off-flavors, these flavor compounds were isolated from milk fat to clarify how to control the process of milk heating. In heated milk products, the contents of lactones were always higher than in unheated products [23,24,25]. It was assumed that in the untreated milk, some precursors are present, and it was confirmed that the corresponding triacylglyceride containing α-5-hydroxy-fatty acids are present [23,24]. Upon heating, the triacylglyceride is hydrolyzed, liberating the hydroxy-fatty acid, and simultaneously lactonized. A possible reaction pathway was suggested earlier [18,26]. Two main aspects were reported to influence the occurrence of hydroxy-fatty acids in the triglycerides of milk: dietary adjustments and the biological origin of the endogenous animal lipid metabolism of the cow [23,27,28,29,30,31,32]. The increase of lactones in AMF induced by high-vacuum sublimation is in agreement with the mentioned literature. The occurrence of lactones as well as the enhancement of lactones in CCB, especially δ-hexadecalactone, has not been described in literature so far.

**Figure 6:**
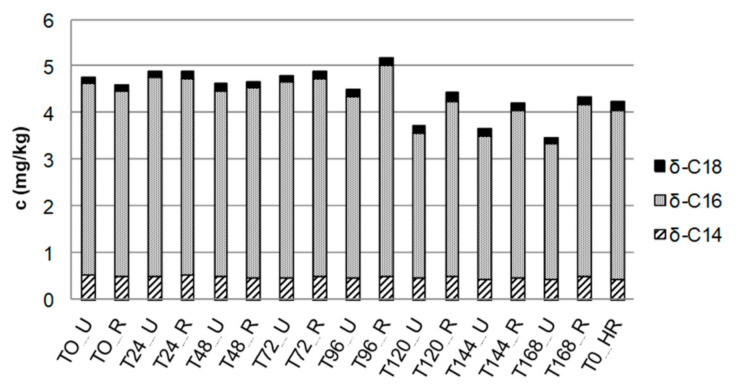
Concentrations of *δ*-tetra- (*δ*-C14), *δ*-hexa- (*δ*-C16), and *δ*-octadecalactone (*δ*-C18) in cocoa liquor of different fermentation times, unroasted (U)/roasted (R)/high roasted (HR), after removal of triglycerides. Concentrations determined by means of LC–MS/MS (SD < 10%).

To investigate the influence of other chocolate ingredients/post-harvest processing parameters to the lactone formation in crumb chocolate, cocoa liquor differing in fermentation time and in roasting degree was examined. The lactone concentration of all studied samples varied between 3 and 5 mg/kg cocoa liquor (Figure 6). Consequently, the amount of lactones in cocoa liquor originates from cocoa butter, which is present in cocoa liquor with a ratio of at least 50%. Neither fermentation time nor roasting enhanced the lactone content (Figure 6).

**Figure 7:**
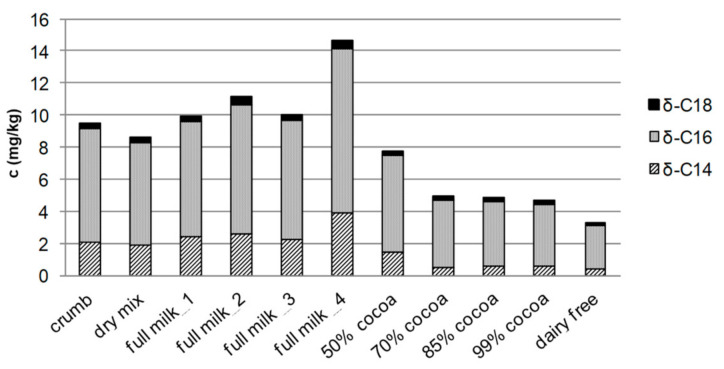
Concentrations of *δ*-tetra- (*δ*-C14), *δ*-hexa- (*δ*-C16), and *δ*-octadecalactone (*δ*-C18) in crumb chocolate, dry mix chocolate (without crumb), four milk chocolate samples (full milk 1–4), and four dairy-free chocolate samples with various contents of cocoa (i.e., 70/85/99%, dairy-free) in fraction Ia (n-pentane extractables without triglycerides). Concentrations determined by means of LC–MS/MS (SD < 10%).

Furthermore, the question arose as to whether the lactone concentration in milk chocolate (AMF and CCB) is generally higher than in dairy-free chocolate, in which only CCB contributes to the lactone content. To answer this question, in addition to the crumb chocolate, six further samples of varying cocoa content, all designated as milk chocolates, were examined (dry mix, full milk no. 1–4, 50% cocoa). Each of the chocolate samples contained at least 28–50% cocoa solids besides different milk components such as clarified butter, (whole/skimmed) milk powder, cream powder, whey product, sweetened condensed milk, or milk sugar. For comparison, four dairy-free chocolate samples, again varying in cocoa content (70%, 85%, and 99% cocoa as well as dairy-free) were analyzed. They consisted only of cocoa mass, low-fat cocoa powder, cocoa butter, and sugar (raw cane) as main ingredients. The total content of the three quantified δ-lactones in the n-pentane extractables without triglycerides was lower than 6 mg/kg (Figure 7). Considering the δ-lactones, δ-tetradecalactone, which mainly originates from different milk ingredients (Figure 3), was found to be higher than 1 mg/kg in all milk chocolates and lower than 1 mg/kg in the dairy-free chocolate samples (Figure 7). Regarding the δ-hexadecalactone, no significant quantitative differences were apparent due to CCB, which was present in every investigated sample. According to these data, it can be concluded that milk chocolates generally have higher lactone contents than dairy-free chocolates (Figure 7).

### 3.2. Sensory Properties of δ-C14/C16/C18-Lactones

According to earlier reports, the flavor attributes of lactones with C10–C18-carbon skeletons were sensorially evaluated in aqueous emulsions as well as in whipped cream. However, these lactones did not impart any creamy sensation in aqueous emulsions; only in whipped cream did δ-tetradecalactone show a creaminess-inducing activity [12]. In the present study, the recognition thresholds for δ-tetradecalactone, δ-hexadecalactone, and δ-octadecalactone were determined in crumb chocolate (Table 2). Lactones of higher chain length (e.g., eicosalactones) were reported to have no retro-nasal or ortho-nasal flavor, and thus, they were not considered in this study [12]. Melted crumb chocolate samples were spiked with the purified lactones, and the panel was asked to describe the sensory impression induced by lactone addition and to determine the recognition threshold by means of a 2-AFC test. Compared to crumb chocolate (control, spiked with the adequate amount of ethanol), the crumb chocolate spiked with δ-tetradecalactone was associated with a stronger creamy flavor; 40 µmol/kg was the lowest concentration at which a significant difference in creaminess was perceptible in the 2-AFC test. The same evaluation was performed for δ-hexadecalactone. Here, the recognition threshold in crumb chocolate was determined at 32 µmol/kg, while for δ-octadecalactone 29 µmol/kg were detected (Table 2). Substitution of δ-tetradecalactone by δ-hexadecalactone or δ-octadecalactone revealed a soapy or buttery sensation for δ-hexadecalactone and an improvement of the fatty mouth feel for δ-octadecalactone in the chocolate. At concentrations above 100 µmol/kg, an “off-flavor” was detected for each lactone. Chocolate spiked with δ-tetradecalactone at 100 µmol/kg evoked a very intensive taste and an almost “sticky” mouth feeling. For δ-hexadecalactone, a strong soapy flavor was perceptible, and for δ-octadecalactone, the fattiness significantly increased (Table 2). However, spiking the crumb chocolate with δ-hexadecalactone or δ-octadecalactone, respectively, led to a significantly accelerated melting behavior in the oral cavity in comparison to the un-spiked crumb chocolate. δ-hexadecalactone provoked a soft film on the palate after melting in the mouth; in contrast, δ-octadecalactone induced a very long-lasting fatty mouth coating effect (Table 2).

## 4. Conclusions

In this study, various δ-lactones were found to influence the overall flavor of crumb chocolate, which was not previously described. However, spiking experiments with individual lactones revealed that δ-tetradecalactone is the one that contributes to the typical creaminess character of crumb chocolate. Furthermore, for the first time, individual recognition thresholds were determined. First, evidence was found that CCB as a vegetable fat source contains a certain concentration of δ-hexadecalactone that can be enhanced by high-vacuum distillation, as well as for the lactone content of AMF, which contributes to the overall lactone content of milk chocolates. It is reasonable to suggest that precursor molecules, e.g., triacylglycerides containing monohydroxyalkanoic acids in the structure, are released and lactonizise or vice versa, during heating.

## Figures and Tables

**Table 1:** Enhancement of creamy flavor by adding the semi-volatile isolate obtained by high-vacuum distillation of cocoa butter (CCB), anhydrous milk fat (AMF), and vegetable fat (VF) at 80/120/150/180 °C. The isolates were suspended in TFLE.

High-Vacuum Distillation	Temperature (°C)			
**Fraction**	**80**	**120**	**150**	**180**
cocoa butter	-	- ^a^	- ^b^	-
anhydrous milk fatb ^c^	+/-	++	++	++
vegetable fat	-	-	-	-

^a^ nutty flavor ^b^ rancid flavor; − no creamy flavor, + slight creamy flavor, ++ strong creamy flavor. ^c^ The enhancement in “creaminess” was determined by twelve panelists using a 2-AFC test (α = 0.05).

**Table 2:** Recognition threshold concentration (*µ*mol/kg) of *δ*-lactones in crumb chocolate.

Lactone	Recognition Threshold (µmol/kg) ^a^	Flavor Quality (Retro-Nasal)	Mouth Coating Effect
*δ*-tetradecalactone	40	creamy	sticky
*δ*-hexadecalactone	32	melted butter	film-forming
*δ*-octadecalactone	29	fatty	long-lasting

^a^ The recognition thresholds were determined by twelve panelists using a 2-AFC test (α = 0.05).

## Data Availability

Data is contained within the article.
